# Supplementary figures and images for: GOLDEN2‐like1 is sufficient but not necessary for chloroplast biogenesis in mesophyll cells of C4 grasses
Source: Plant J. 2023 Oct 26;117(2):416–31. doi: 10.1111/tpj.16498 (PMC10953395; doi:10.1111/tpj.16498)

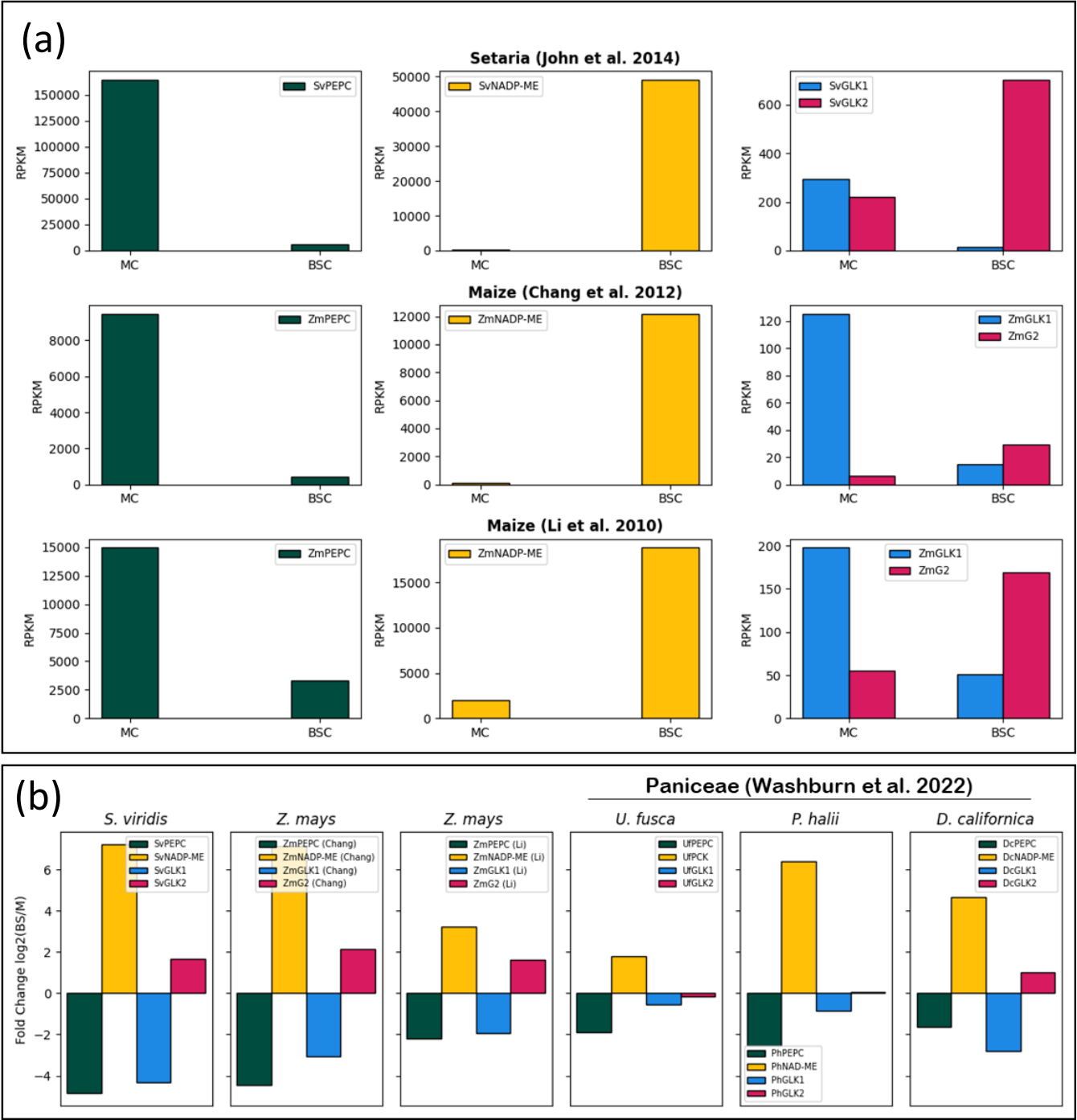

**Figure S1.**

Supplement: Supplementary file 1 — Figure S1. GLK transcript accumulation in bundle sheath and mesophyll cells. (a) Gene expression in reads per kilobase million (RPKM) in mesophyll (MC) and bundle sheath cells (BSC) of setaria (SvPEPC, SvNADP‐ME, SvGLK1, and SvGLK2) and maize (ZmPEPC, ZmNADP‐ME, ZmGLK1, and ZmG2). Maize data were published by Li et al. (2010) and Chang et al. (2012), and setaria data were published by John et al. (2014). PEPC and NADP‐ME transcripts accumulate specifically in mesophyll and bundle sheath cells, respectively, and thus act as markers for cross‐contamination of RNA samples between cell types. As such, mesophyll contamination of bundle sheath transcriptomes was 22% (Li et al), 4.5% (Chang et al), and 3.5% (John et al), whereas bundle sheath contamination of mesophyll transcriptomes was 10.7% (Li et al), 7% (Chang et al), and 6.6% (John et al). Disregarding the Li et al. (2010) data because of the high level of cross‐contamination, GLK1 transcript levels were higher in mesophyll than bundle sheath cells by 8‐fold in maize and 21‐fold in setaria, whereas GLK2 transcript levels were higher in bundle sheath than mesophyll cells by 4.8‐fold in maize and 3.2‐fold in setaria. (b) Log2 fold change between bundle sheath and mesophyll transcripts for S. viridis (John et al., 2014), Z. mays (Chang et al., 2012; Li et al., 2010), and three different species of Paniceae grasses (Urochloa fusca, Panicum hallii, and Digitaria californica) (Washburn et al. 2022). All species carry out C4 photosynthesis, but each uses a different decarboxylation pathway in the bundle sheath cells. PEPC was used as a mesophyll cell marker in each case and genes encoding the relevant decarboxylation enzyme (i.e., NADP‐ME, PCK, and NAD‐ME) were used as bundle sheath cell markers. [file TPJ-117-416-s003.pdf]

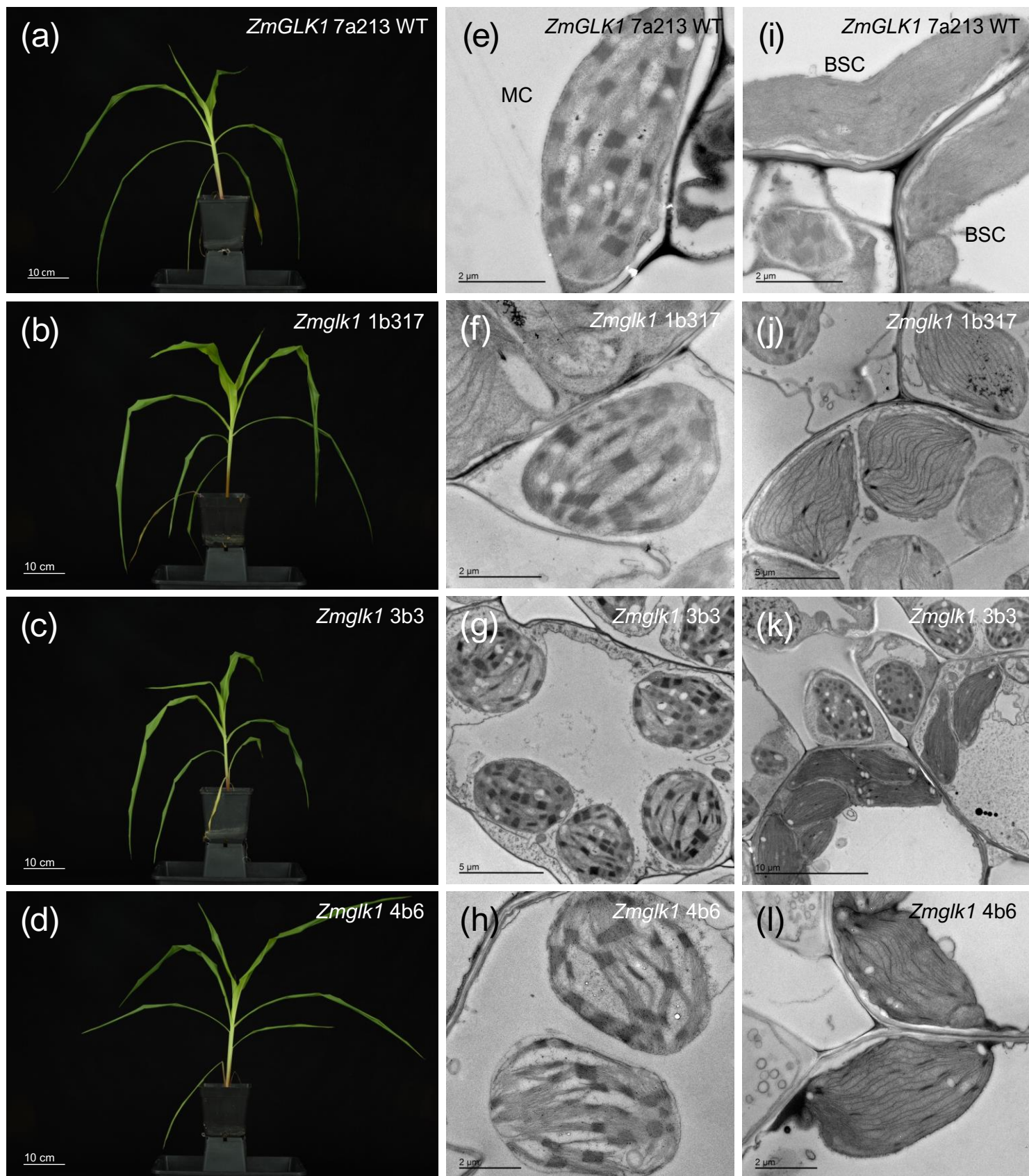

**Figure S2.**

Supplement: Supplementary file 2 — Figure S2. Phenotypic characterization of three independent Zmglk1 mutant lines. (a–d) Whole plant phenotype 30 days after sowing. Scale bars = 10 cm. (e–h) Transmission electron microscopy (TEM) images showing mesophyll chloroplast ultrastructure. Scale = 2 μm, 2 μm, 5 μm, and 2 μm, respectively. (i–l) TEM images showing bundle sheath chloroplast ultrastructure. Scale bars = 2 μm, 5 μm, 10 μm, and 2 μm, respectively. [file TPJ-117-416-s009.pdf]

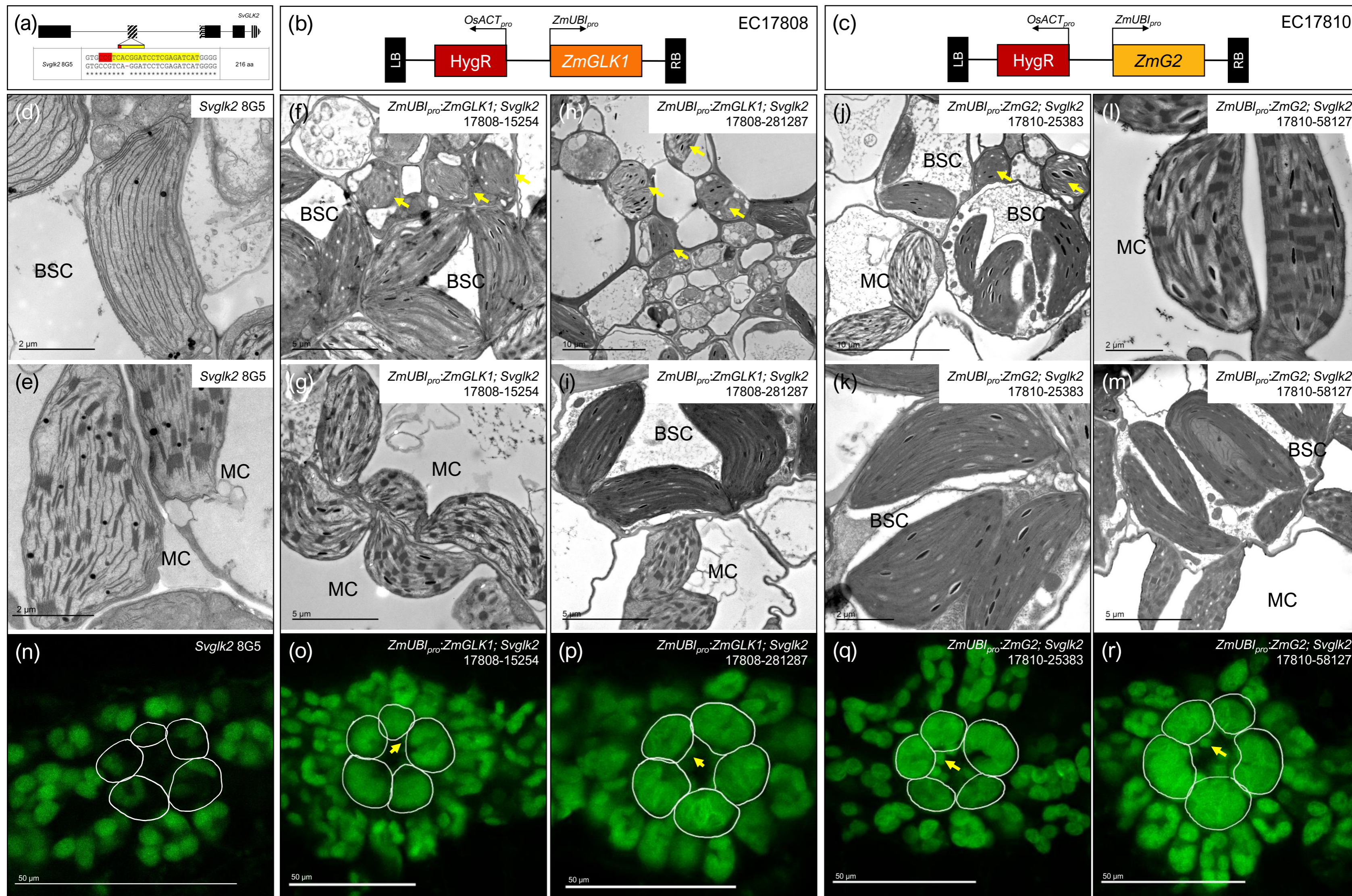

**Figure S5.**

Supplement: Supplementary file 5 — Figure S5. Phenotypic characterization of Svglk2 mutant lines complemented with ZmGLK1 or ZmG2. (a) Schematic of the gene‐edited Svglk2 mutant allele. (b,c) Schematic of the constructs used to express ZmGLK1 (b) or ZmG2 (c) in the Svglk2 background. HygR depicts the hygromycin phosphotransferase gene and OsACT pro and ZmUBI pro represent the constitutive rice actin and maize ubiquitin promoters, respectively. LB and RB refer to left and right borders. (d–m) Transmission electron microscopy images showing mesophyll (MC) and bundle sheath (BSC) cell chloroplast ultrastructure in the mutant line (d,e) and in lines overexpressing ZmGLK1 (f–i) or ZmG2 (j–m) in the mutant background. Scale bar sizes are indicated on each image. Panels d & e are the same images as Figure 3g,h). (n–r) Confocal images of leaf cross sections from the mutant (n) and from lines overexpressing ZmGLK1 (o,p) or ZmG2 (q,r) showing the morphology of chloroplasts. Bundle sheath cells are outlined in white. Scale bars = 50 μm. Yellow arrows indicate ectopic chloroplast formation in vascular cells [file TPJ-117-416-s001.pdf]

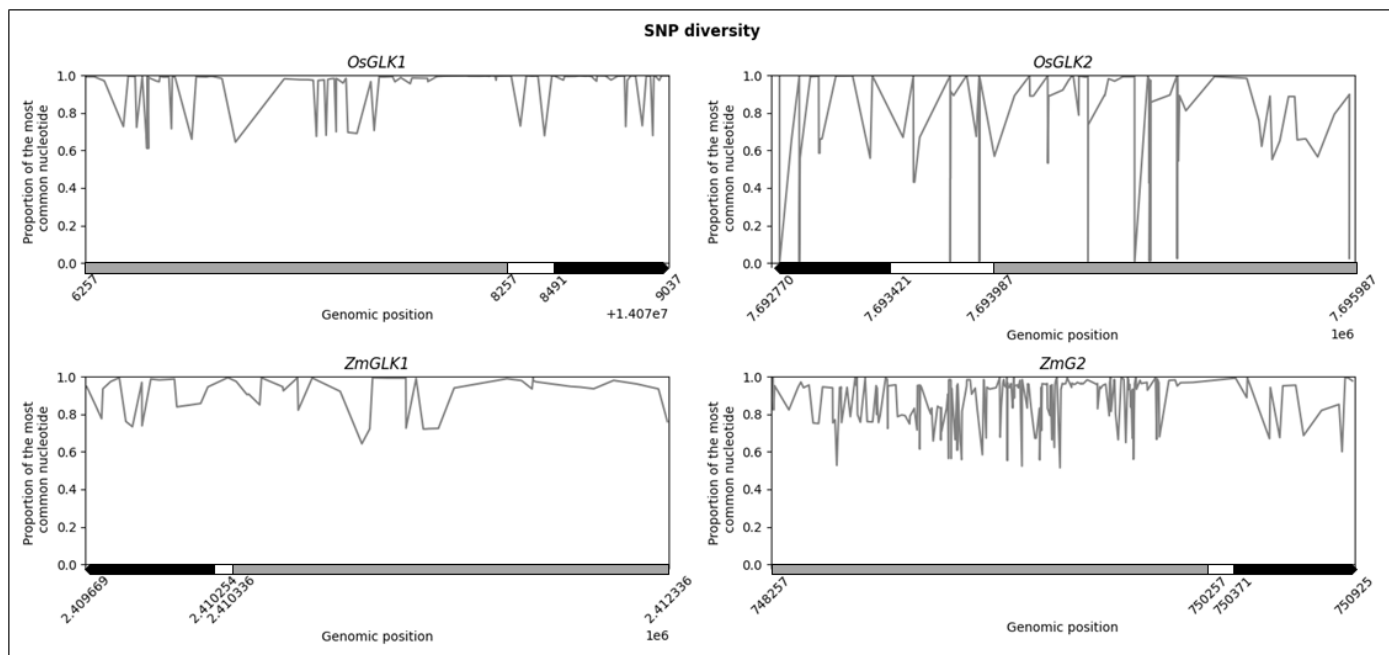

**Figure S6.**

Supplement: Supplementary file 6 — Figure S6. Sequence variability in regulatory regions of GLK genes. The SNPs from 3025 variants of rice (SNP seek, IRRI) and 1210 variants of maize (Maize SNPDB) were used to calculate the frequency of each nucleotide for each SNP position. The nucleotide with a higher prevalence in each position is plotted on the graphs for OsGLK1, OsGLK2, ZmGLK1, and ZmG2. The 2Kb upstream of the transcription start site is shown in gray, the 5’‐UTR in white, and the first exon in black. [file TPJ-117-416-s008.pdf]
