# Supplementary material for: GOLDEN2‐like1 is sufficient but not necessary for chloroplast biogenesis in mesophyll cells of C4 grasses
Source: Plant J. 2023 Oct 26;117(2):416–31. doi: 10.1111/tpj.16498 (PMC10953395; doi:10.1111/tpj.16498)
Supplement: Supplementary file 4 — Figure S4. DNA binding motifs in regulatory regions of GLK genes. (a) Schematics of maize, setaria, and rice GLK genes showing binding sites revealed from ATAC assays with leaf RNA. Data was retrieved from https://epigenome.genetics.uga.edu/PlantEpigenome/index.html. The yellow shading highlights the 800 bp upstream of the transcription start site plus the 5’‐UTR regions. (b) Table showing the number of DNA binding motifs found in regulatory regions of each gene. Plant non‐redundant motifs (JASPAR) were used to screen the above‐mentioned regions of each gene, using the FIMO tool with a P value of <0.0001. The photosynthesis‐related/light responsive motifs (BPC, ABI3‐like, GT‐1, GT‐4, CNA, MYB‐B, STZ, bZIP/bHLH(G‐box), GATA, MYB‐like/I‐box, TCP, TGA, and WRKY) were as described by Sing et al. (2023) and rice and maize GLK‐specific binding sites were as described in Tu et al. (2022). (c,d) Schematics of 800 bp upstream of the transcription start site plus the 5’‐UTR regions for each gene, showing the position of all predicted DNA binding motifs (c) and of known light‐responsive motifs (d). The 5’‐UTR is shown in white and promoter sequence is in gray. [file TPJ-117-416-s006.pdf]

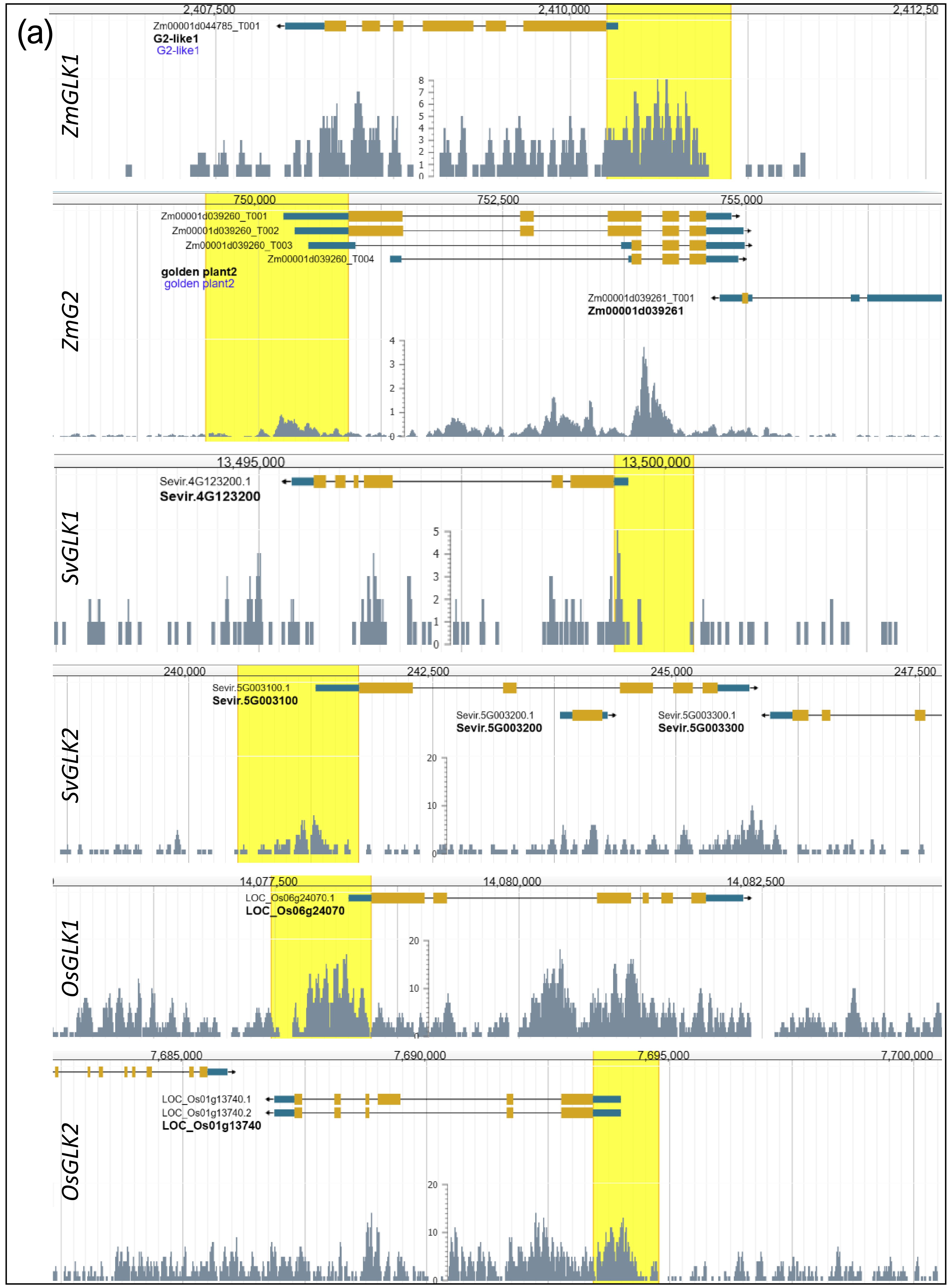

(b)

Motif	ZmGLK1	ZmG2	SvGLK1	SvGLK2	OsGLK1	OsGLK2
ABI3-like	1	3	2	0	0	2
BPC	1	47	1	81	19	133
GATA	3	9	17	3	0	4
MYB-B	3	1	2	0	6	2
TGA	1	0	0	0	0	0
WRKY	20	0	0	21	0	0
bZIP/bHLH (G-box)	0	8	1	3	0	8
MYB-like/I-box	0	10	10	3	9	7
TCP	0	1	0	0	1	1
GT-1	0	0	0	0	0	1

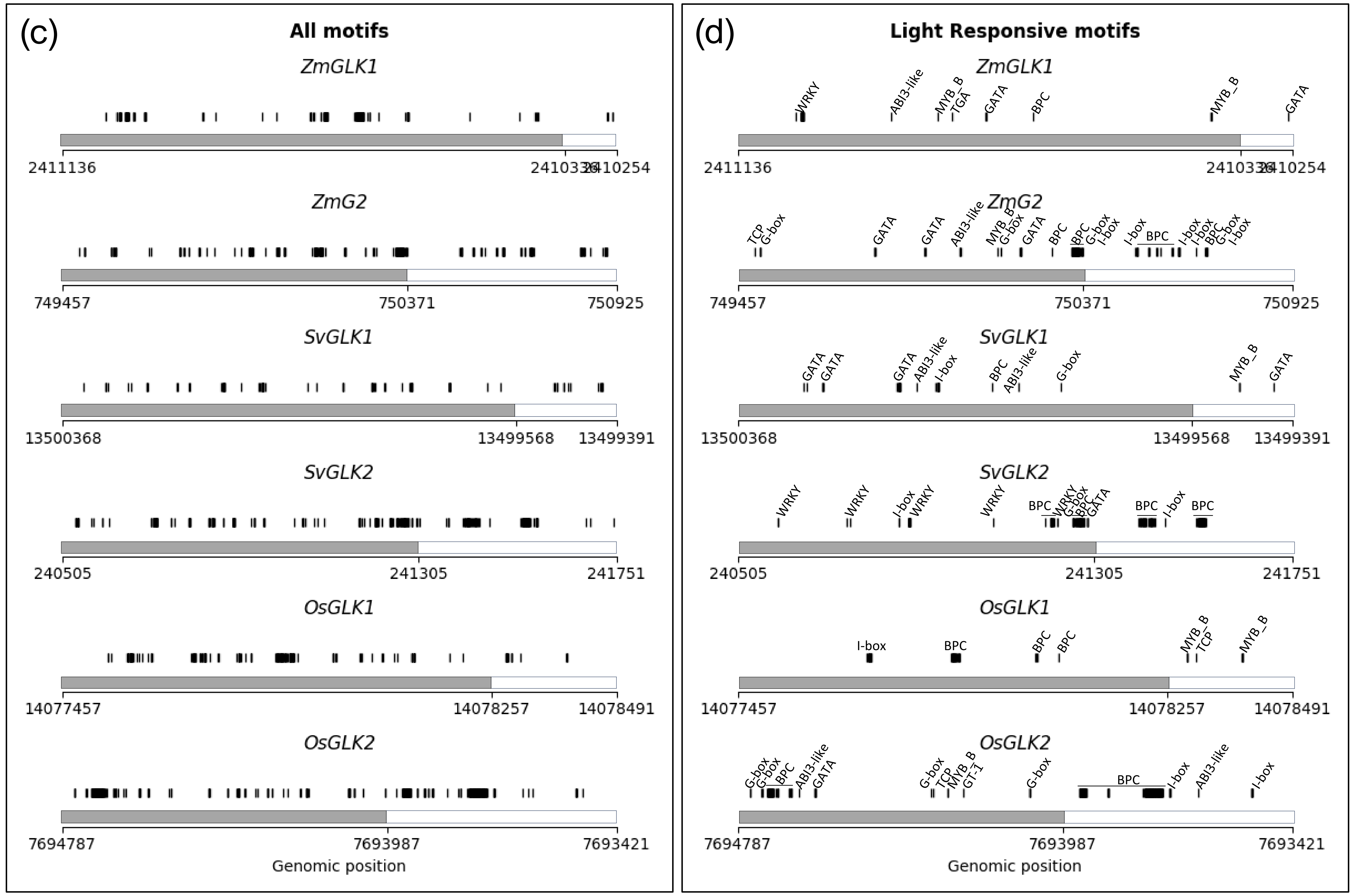

Figure S4.
