# Supplementary material for: GOLDEN2‐like1 is sufficient but not necessary for chloroplast biogenesis in mesophyll cells of C4 grasses
Source: Plant J. 2023 Oct 26;117(2):416–31. doi: 10.1111/tpj.16498 (PMC10953395; doi:10.1111/tpj.16498)
Supplement: Supplementary file 3 — Figure S3. Comparison of GLK sequences. (a) Phylogenetic reconstruction of the GLK gene family. Protein sequences of the extensively characterized GLK members ZmGLK1, ZmG2, OsGLK1, and OsGLK2 were used to create a HMMER profile that was used as a query to search for homologs in whole protein databases of 11 species (Zea mays, Setaria viridis, S. italica, Panicum virgatum, Sorghum bicolor, Oryza sativa, Brachypodium distachyon, Vitis vinifera, Arabidopsis thaliana, Marchantia polymorpha, and Physcomitrium patens). The presence of the MYB domain in those sequences was confirmed using InterproScan, and only the sequences containing the conserved AREAEAA domain characteristic of the GLK subfamily were selected. The resulting 25 amino acid sequences were aligned using Clustal Omega. Phylogenetic reconstruction was performed using Bayesian inference on Mr. Bayes using Poisson with fixed substitution rates as a model. Two independent MCMC runs with 1 000 000 generations were performed and no further improvement of log‐likelihood of prior probabilities was observed. The tree was visualized on FigTree. Clade support on each node was assessed using Bayesian posterior probabilities. Members of the GLK1 subgroup are depicted in green and GLK2 in blue. (b) Amino acid sequence alignment showing conservation between ZmGLK1, SvGLK1, ZmG2, and SvGLK2. The diagonal lines show the HLH associated with MYB or MYB‐related proteins; the AREAEAA conserved site is depicted in horizontal lines and the last exon that contains the GCT‐box domain is depicted in vertical lines. [file TPJ-117-416-s005.pdf]

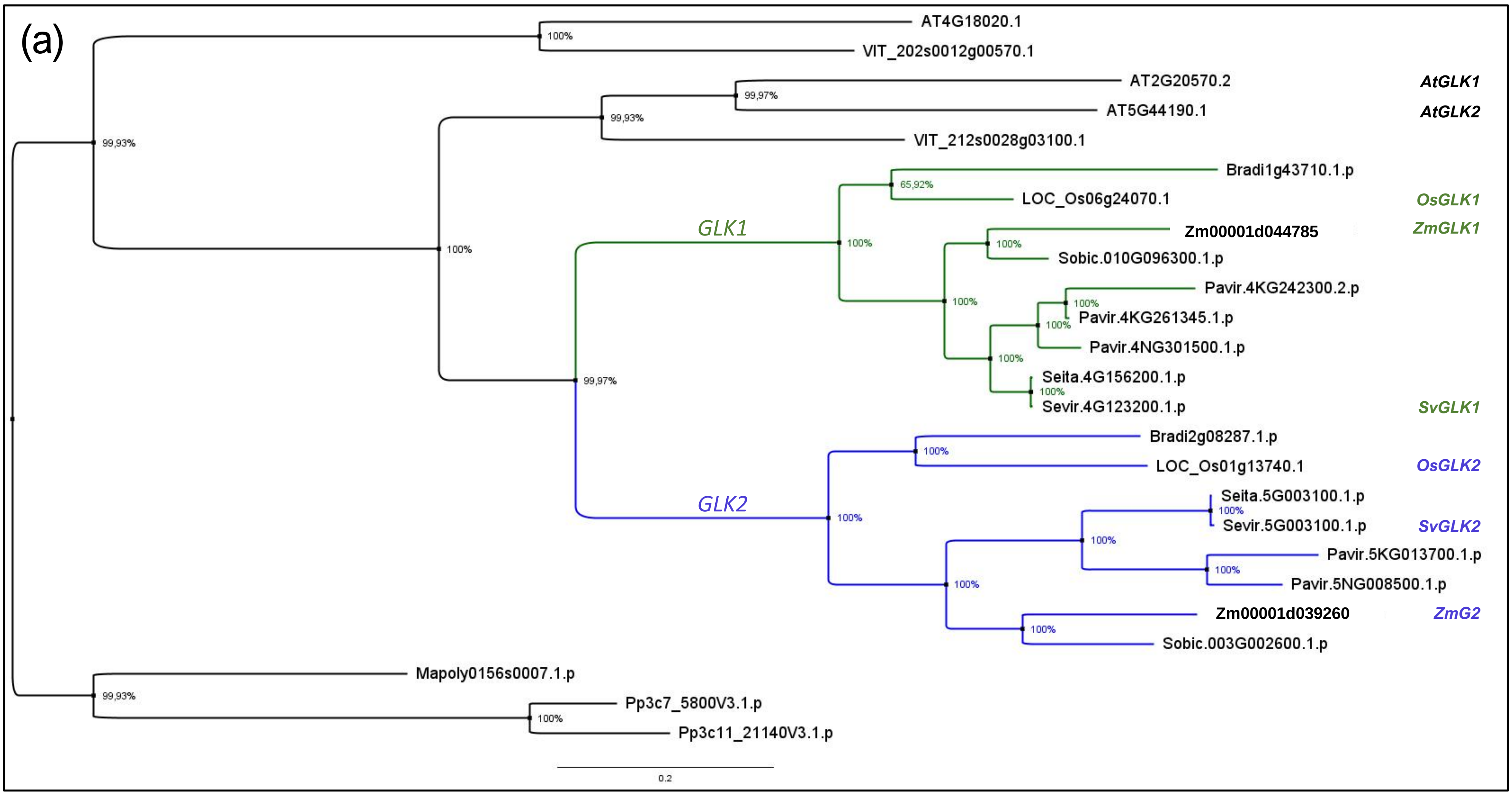

(b)

```
ZmGLK1          MLAVSPSPV------RCADAEECGGGGASKEMEETAVGPVSDSDLDFD---FTVDDIDFGDF--FLRLDDGDDALPGLEVDPAEIVFADFEAIATAG-----GDGGVTDQEVPSVLPFAD
SvGLK1          --MLAVSPV------RCADADECGGGAGM---EMEEIGPV--SDLDFD---FTVDDIDFGDF--FLRLEDGD-ALPDLEVDPAEI-FTDFEAIA--T-----GSDGVMDQEVPSVQPLTD
ZmG2            --MLEVSTLRGPTSSGSKAEQHCGGGGGFVGDHHVV--FPTSGDCFAMVDDNLLDYIDFS--CDVPFFDADGDILPDLEVDTTELL-AEFSSTPPADDLLAVAVFGADDQPAAAVAQE--
SvGLK2          --MLEVSSLRSPSKA--------ADQRGFVGDHVGF--PTSAGTDDFMVDDNLLDYIDFSSCCDMPFFDADGDILPDLEVDPTESLLAEFSSPE-TSPAAADGGGGAEAKPPDEKQEQVA
                   :  * :              ..  .    .         .          :* ***.    .  :: ..  **.**** :*   ::*.:          .  *.  :

ZmGLK1          AAHIGAVDPCCGVLGEDNDAACADVEEGKGECDHADEVAAAGNNNSDSGEAGCGGAFAGEKSPSSTASSSQEAESRRKVSKKHSQGKKKAKVDWTPELHRRFVQAVEELGIDKAVPSRIL
SvGLK1          AVHLDALDPCSVVLGEDNTT-RADVEEGKGECNHAEEVVPGNGDF----GGGGGTVLAEEKSPSSTTSSSQEAESRHKSSSKHSHGKKKAKVDWTPELHRRFVQAVEQLGIDKAVPSRIL
ZmG2            -KPSSSLEQTC---GDDKGVAVAAARRKL--QTTTTTTT---TEEEDSSPAGSGAN---K-SS---ASAEGHSSKKKSAGKNSNGGKRKVKVDWTPELHRRFVQAVEQLGIDKAVPSRIL
SvGLK2          AKTAAAEETKA---DDDEKTAAGGVVVTR--KRNGDEVT---TTEEDSAGAGAGSD---TKSS---AAAGGHSKKKASAGKDS-NGKRKVKVDWTPELHRRFVQAVEQLGIDKAVPSRIL
                     : :  .   .:*: .  . .            ..           .* *       *    :::  .:..: . ...   **:*.*****************:************

ZmGLK1          EIMGIDSLTRHNIASHLQKYRSHRKHMLAREVEAATWTTHRRPMYAA--PSGAV--KRPDSNAWTVPTIGFPPPAG--TPPRPVQHFGRPLHVWGHPSPTPA-----VESPRVPMWPRHL
SvGLK1          EIMGIDSLTRHNIASHLQKYRSHRKHMLAREAEAASWTQRRQ-MYAAGGASTAV--KRPDSNAWTVPTIGFPPPPPPPPPPPHPMQHFGRPLHVWGHPT--PG-----VESPRVPMWPRHL
ZmG2            EIMGTDCLTRHNIASHLQKYRSHRKHLMAREAEAATWAQKRH-MYAPP-APRTTTTTDAARPPWVVPTT---------I-GFPPPRFCRPLHVWGHPPPHAAAAEAAAATPMLPVWPRHL
SvGLK2          EIMGIEGLTRHNIASHLQKYRSHRKHLMAREAEAATWAQKRQ-LYAAAPVARKVEAAAAAGPPWVVPTIGFPPPPPPAM-APPPPPFCRPLHVWGHPPTAAVE----APATMLPVWPRHL
                **** : *******************::***.***:*: :*: :**       .         *.***              *   * *********          . :  :*:*****

ZmGLK1          APRAPPPPPWAPPPPADPASFWHHAYMRQGPAA--HMPDQVAVTPCVAVPMAAARFPAPHVRGSLPWPPPMYRPLV--PPALAGKSQQDALFQLQIQPSSESIDAAIGDVLTKPWLPLPL
SvGLK1          VPRAPT-PPWAPPPPSDP-AFWHHPYMRGPA----QMPG--QMAPCMAVPMPAARFPAPPVRGVLPCPPPMYRPLV--PPVLANKSQQDAQLQLQTQPSSESIDAAIGDVLSKPWLPLPL
ZmG2            APPRHL-APWAHPTPVDP-AFWHQQYSAA-RKWGPQAAAVTQGTPCVPLPRF-----P--VPHPIYSRPAMVPPP------PSTTKLAQLHLELQAHPSKESIDAAIGDVLVKPWLPLPL
SvGLK2          APPRP----W-----VDP-GYWHQQYNAGRTKWAPPQAAVTQGTPCVPPPPAMVQLPR--FPHPMYSRPPVMVPPPPPPPTTTTTKLAELQLQLDAHPSKESIDAAIGDVLVKPWLPLPL
                .*       *      ** .:**: *                 :**:  *          .   :   * :  *        : ..  :  ::*: :**.*********** ********

ZmGLK1          GLKPPSVDSVMGELQRQGVANVPQACG*-------
SvGLK1          GLKPPSVDSVMDELQRQGVADVPPACG*-------
ZmG2            GLKPPSLDSVMSELHKQGVPKIPPAAATTTGATG*
SvGLK2          GLKPPSLDSVMSELHKQGIPKIPPASSTG*-----
                ******:****.**::**: .:* *..
```

Figure S3.
