# Supplementary material for: GOLDEN2‐like1 is sufficient but not necessary for chloroplast biogenesis in mesophyll cells of C4 grasses
Source: Plant J. 2023 Oct 26;117(2):416–31. doi: 10.1111/tpj.16498 (PMC10953395; doi:10.1111/tpj.16498)
Supplement: Supplementary file 7 — Figure S7. Alignment of GLK gene promoter and 5’‐UTR regions. (a, b) GLK1 (b) and GLK2(a) sequences (800 bp upstream of the transcription start site (TSS) plus the 5’‐UTR) aligned using MAFFT (Katoh et al., 2019) and visualized using JALview (Waterhouse et al., 2009). Conserved bases are indicated by blue shading, the predicted TATA box by the red squares, actual (rice—Murray et al., 2022), or predicted TSS by orange squares and putative uORFs by yellow squares. (c) Amino acid sequences of the predicted uORFs in (a) & (b). [file TPJ-117-416-s007.pdf]

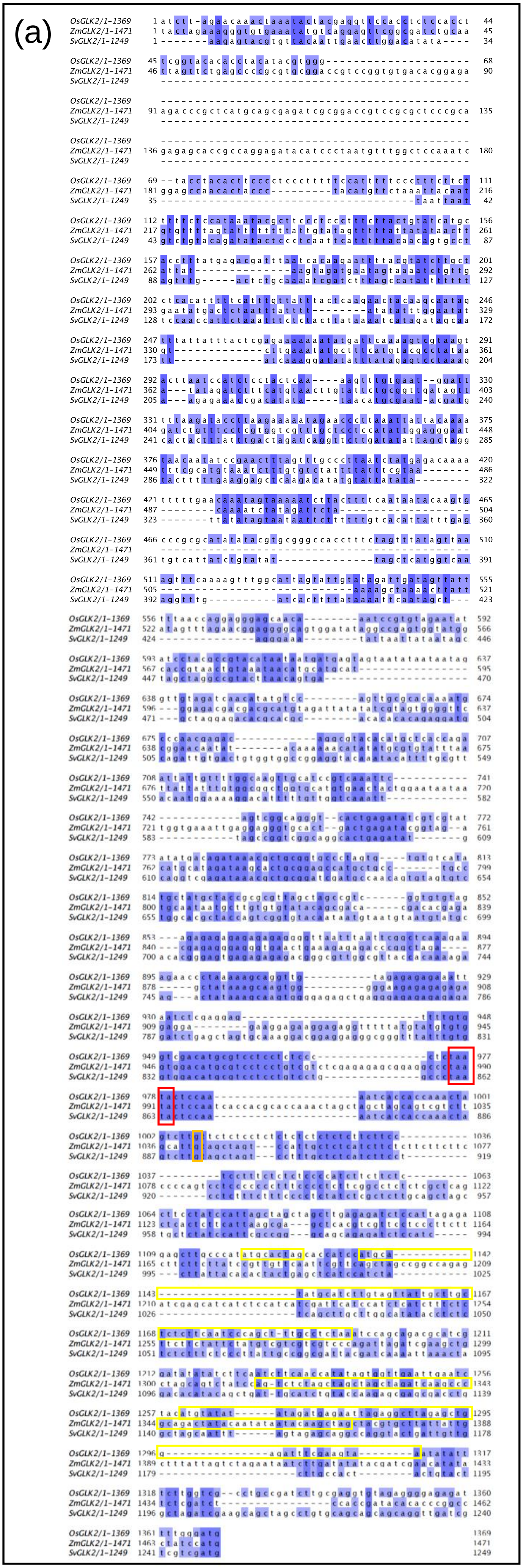


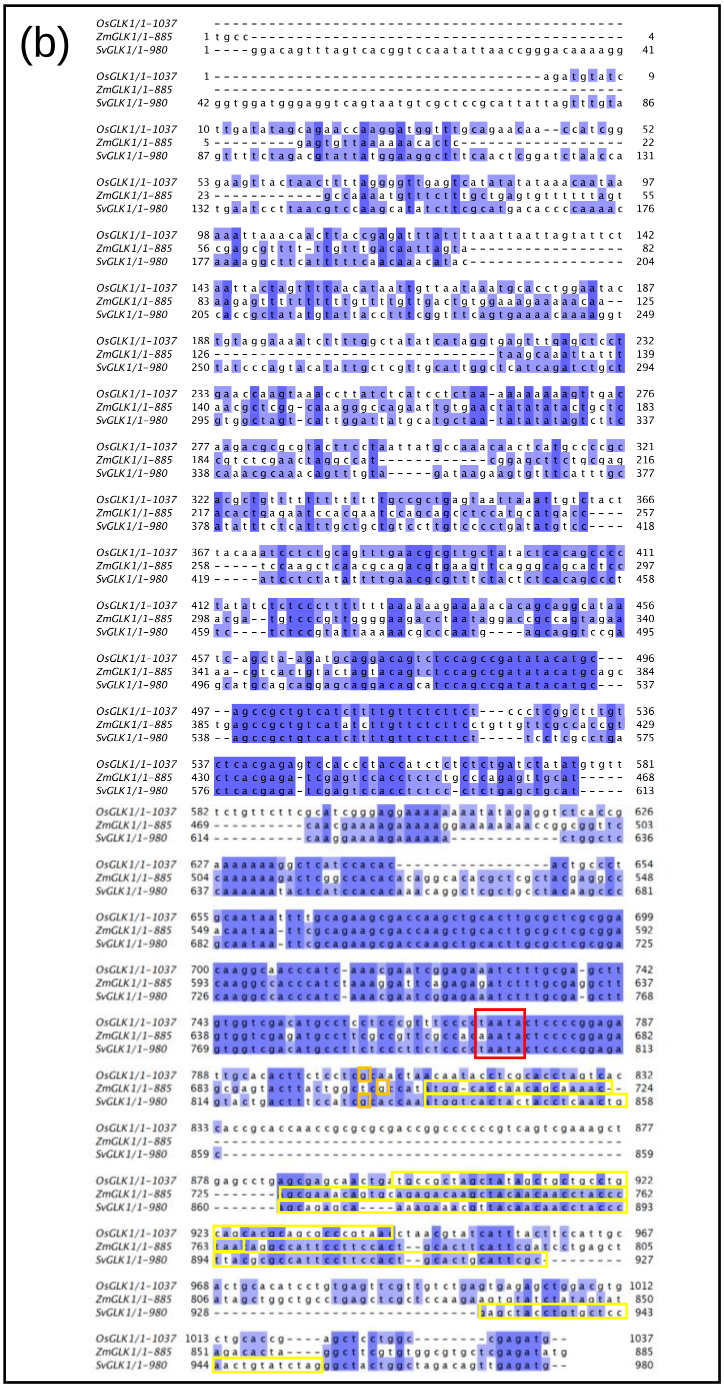


(c)

OsGLK1: MPLAIAAACSTQRP*

OsGLK2: MH*

MHMHLVVIACSLQSQLCL*

MRIRGLELEISK*

ZmGLK1: MAPTAKQRNSAETSYNNLP*

ZmGLK2: MSSSSQIRSKLLAVYPVSS*

SvGLK1: MVTTTSTAAEQKKRYNNLPLRAIPSTAL
HSRATCAPTVSRATG*

# Figure S7.
